# Supplementary material for: Network dynamics-based cancer panel stratification for systemic prediction of anticancer drug response
Source: Nat Commun. 2017 Dec 5;8:1940. doi: 10.1038/s41467-017-02160-5 (PMC5717260; doi:10.1038/s41467-017-02160-5)
Supplement: Supplementary file 1 — Supplementary Information [file 41467_2017_2160_MOESM1_ESM.pdf]

Supplementary Note 1

State-transition logic for Boolean network modeling

We employed Boolean logic network modeling, in which only logical relationships between the activities of network nodes are considered. To transform the static map shown in Figure 1b into a dynamical model, which accounts for different scenarios or a set of events leading to distinct cellular states, we defined state transition logic based on experimental evidences. We transformed the state transition logic into a weighted sum logic with the weight (activation or inhibition) of each link and base level (i.e. the basal activity level) of each node (see Supplementary Data 6 for details).

Each node in the Boolean network model can have either ON or OFF state at a specific time step, which represents the active or inactive state of the molecule, respectively. Each node receives inputs from one or more nodes and has a distinct base level, depending on network condition, such as node deletion. So the state of a node in the next time step depends on its current state and base level as follows:

$$\mathrm{S_i}(\mathrm{t}+1) = \begin{cases} 1, & \text{if} \quad \sum_{\mathrm{j}} (a_{\mathrm{ij}}\mathrm{S_j}(t) + \mathrm{b_i}) > 0 \\ 0, & \text{else if} \quad \sum_{\mathrm{j}} (a_{\mathrm{ij}}\mathrm{S_j}(t) + \mathrm{b_i}) < 0 \\ \mathrm{S_i}(\mathrm{t}), & \text{else if} \quad \sum_{\mathrm{j}} (a_{\mathrm{ij}}\mathrm{S_j}(t) + \mathrm{b_i}) = 0 \end{cases}$$

where $\mathrm{S_i}(t)$ is the state of target node i at time step t and a_{ij} is an interaction weight that accounts for either activating or suppressing interaction between the target node i and input node j in the network. Each interaction weight depends on the current states of input nodes. The base level of each node (b_{i}) is the value determining the state value of the node in the absence of input.

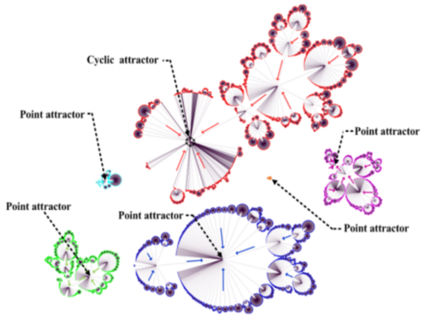

Type	ATM	p53	Mdm2	Mdmx	Wip1	cyclin G	PTEN	p21	AKT	cyclin E	Rb	E2F1	p14ARF	Bcl-2	Bax	caspase	Basin size (Ratio)	Cell state
Cyclic	■		■		■				■	■		■			■		42840(0.65)	A
	■	■			■				■	■		■						
	■	■		■	■	■	■	■	■	■		■			■			
			■	■	■	■	■	■	■						■			
			■	■	■							■						
Point		■	■		■	■	■		■	■		■			■	■	13824(0.21)	D
Point		■	■		■	■	■		■	■		■			■	■	4928(0.08)	D
Point		■	■		■	■	■		■	■		■			■	■	3032(0.05)	D
Point			■	■					■	■	■						882(0.01)	P
Point			■						■	■	■						30(0.0004)	P

Supplementary Figure 1. Illustrating the definition of a cellular response phenotype in the attractor landscape of p53 network. Cellular response phenotypes including cell proliferation, cell cycle arrest and cell death can be represented by attractor states, and their relative ratios are measured by calculating the basin of attraction. In this example, the major cellular phenotype is cell cycle arrest since the major attractor state (cyclic attractor, basin size: 42,840) shows an oscillatory activation of p21 (ON/OFF), oscillatory activation of Cyclin E (ON/OFF) and inactivation of caspase (OFF) (P: cell proliferation, A: cell cycle arrest, D: cell death).

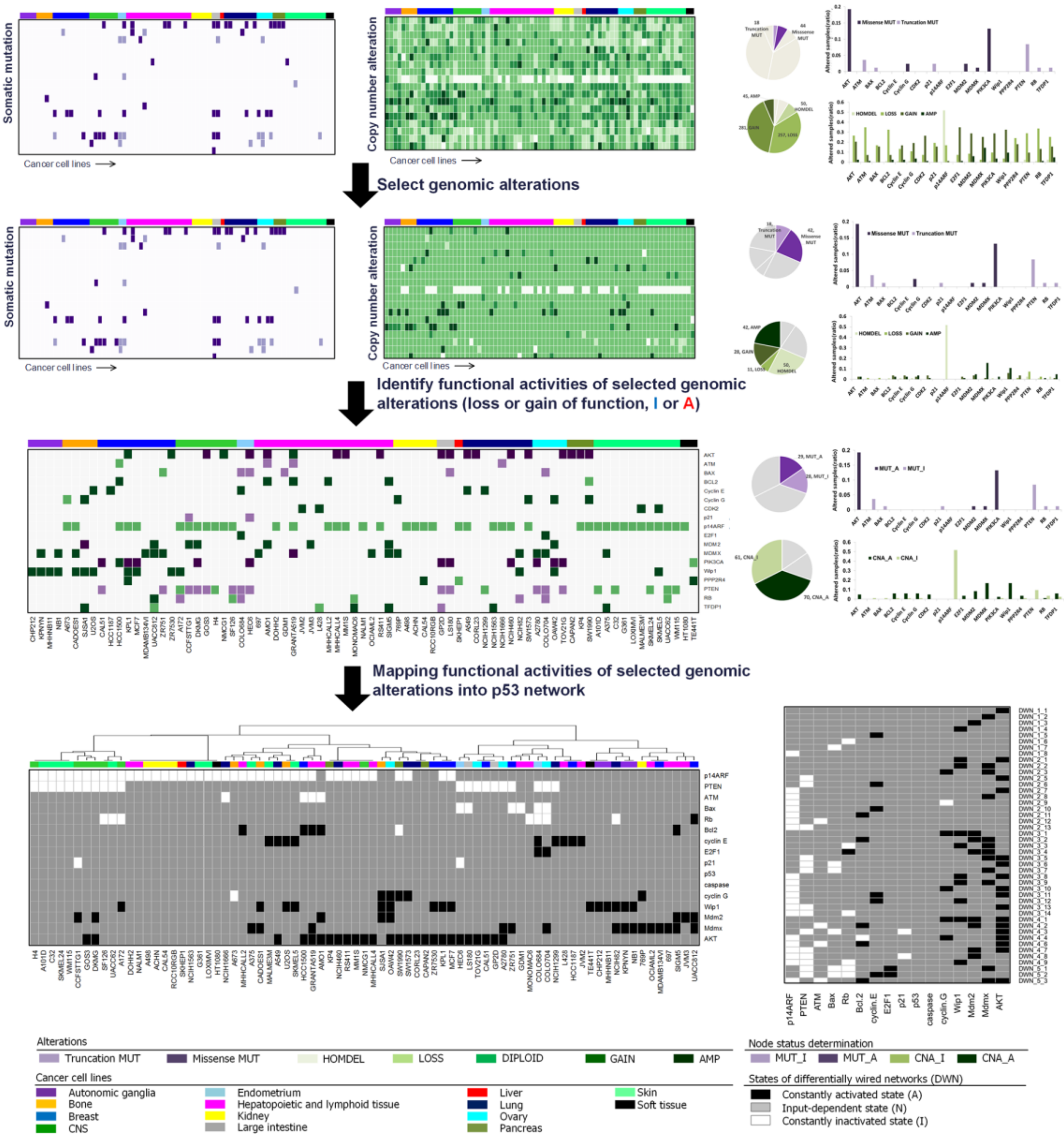

Supplementary Figure 2. The procedure of mapping the genomic alterations onto network. Copy number alterations and somatic mutations are considered as genomic alterations. A few hundred candidate functional events associated with the p53 network were selected from thousands of genomic alterations. The selected alterations tend to involve well-known oncogenes and tumor suppressors. For each data type, candidate functional alterations selected were evaluated for whether it is gain of function or loss of function, and then denoted as constantly activated (A) or constantly inactivated (I), depending on its alteration type in a given cancer cell line. These alterations were then projected onto the nominal p53 network model. The node status in p53 network is determined in a ternary fashion, such that node activity is either constantly activated (A), constantly inactivated (I) or input-dependent (N). In total, 45 distinct differentially wired p53 networks were reconstructed. And they involve one to four perturbations. Cancer cell lines that have the same node activity profile are mapped to an identical single network.

Genomic alterations -> Functional genomic alterations

Mutation	Functional Impact Score	Functional mutation
nonsense		Selected
missense	High or Medium	Selected

Copy number alteration (CNA)	mRNA expression (by Z score)	Functional CNA
HOMDEL		Selected
LOSS	DOWN(Z score <-2)	Selected
GAIN or AMP	UP(Z score >2)	Selected

Functional genomic alterations -> Network perturbations

Functional genomic alterations	Function	Node status
nonsense		Constantly inactivated
missense	Oncogene (Tumor suppressor)	Constantly activated(inactivated)
HOMDEL or LOSS		Constantly inactivated
GAIN or AMP		Constantly activated

Supplementary Figure 3. From genomic alterations to network perturbations. To include genomic alterations that were likely functional, missense mutation that has a high or medium functional impact score from MutationAssessor (http://mutationassessor.org) was selected, as well as nonsense mutation. Also, for the selection of genes with copy number alteration, z scores were precomputed from the expression values. The z scores for mRNA expression were determined for each sample by comparing a gene's mRNA expression to the distribution in a reference population that represents typical expression of the gene. Hence, when compared to wild-type cases, genes with copy number alterations that have z scores of $z > 2$ or $z < -2$ were defined as having concordant changes in mRNA expression levels. Next, for mapping the selected genomic alteration onto the nominal p53 network, functional outcome of each selected alteration was determined by its alteration and functional type in a given cancer cell line, such that a gene (protein) with the alteration was either constantly activated (A) or constantly inactivated (I).

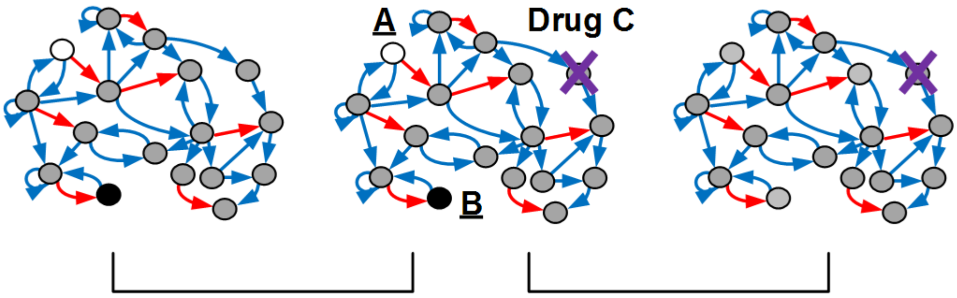

Comparison of major cellular phenotypes

Genomic alterations	Inhibitory treatment	Major cellular phenotype			
		Case 1	Case 2	Case 3	Case 4
A(I) and B(A)	NO	P	P	P	P
NO	Drug C	P	A	D	P
A(I)	Drug C	A	A	A	A
B(A)	Drug C	P	P	P	A
A(I) and B(A)	Drug C	P	P	D	D

Case 1: no critical target and no critical determinant
Case 2: no critical target and critical determinant (B(A))
Case 3: critical target (Drug C) and no critical determinant
Case 4: no critical target (Drug C) and no critical determinant (A(I) and B(A))

Supplementary Figure 4. Examples of drug response profiling to identify a critical target and critical determinant. We investigated whether a given targeted node/link is a critical target by comparing the major cellular phenotypes before and after the drug-induced perturbation. If they are the same (if they are different), the gene targeted by the given drug is not a critical target (the targeted gene is not a critical target). We also identified critical determinant, which is a minimal set of genomic alteration that determines a major cellular phenotype for a given inhibitory treatment. Specifically, for a given inhibitory treatment, the presence or absence of critical determinant is determined by comparing major cellular phenotype of cancer cell line network with that of normal network that has no alteration. If they are the same, we consider genomic alterations in the network do not contribute to determining major cellular phenotype in response to the specific inhibitory treatment (i.e., there is not critical determinant in the network). On the other hand, if they are different, critical determinant can be found from the perturbation results based on network dynamics (Supplementary Data 3, A(I): node A is constantly inactivated (white circle), B(A): node B is constantly activated (black circle)).

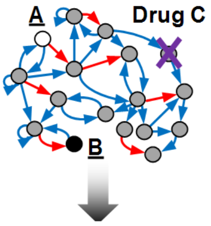

Genomic alterations	Inhibitory treatment	Major cellular phenotype
A(I) and B(A)	NO	P
NO	Drug C	P
A(I)	Drug C	A
B(A)	Drug C	A
A(I) and B(A)	Drug C	D

critical target (Drug C) and no critical determinant (A(I) and B(A))

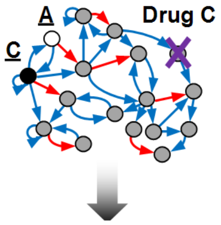

Genomic alterations	Inhibitory treatment	Major cellular phenotype
A(I) and C(A)	NO	P
NO	Drug C	P
A(I)	Drug C	A
C(A)	Drug C	D
A(I) and C(A)	Drug C	D

critical target (Drug C) and critical determinant (C(A))

Supplementary Figure 5. Network dynamics-based analysis reveals critical determinant, which is the minimal set of genomic alterations that determine the major drug response phenotype. If a specific drug perturbation (Drug C) induces strong cell death in two distinct networks, in which node A is constantly inactivated (white circle in network), it is self-evident that node A may be commonly associated with the death efficacy of Drug C. However, the critical determinants of two distinct networks are different (left: A(I) and B(A), right: C(A)). It indicates these different genomic alteration(s) affect their cancer network dynamics which determines a drug response phenotype. Therefore, to understand complex cellular processes and identify the source of variability in drug response, we need to investigate the network dynamics at the system level. (A(I): node A is constantly inactivated (white circle), B(A): node B is constantly activated (black circle), C(A): node C is constantly activated (black circle))

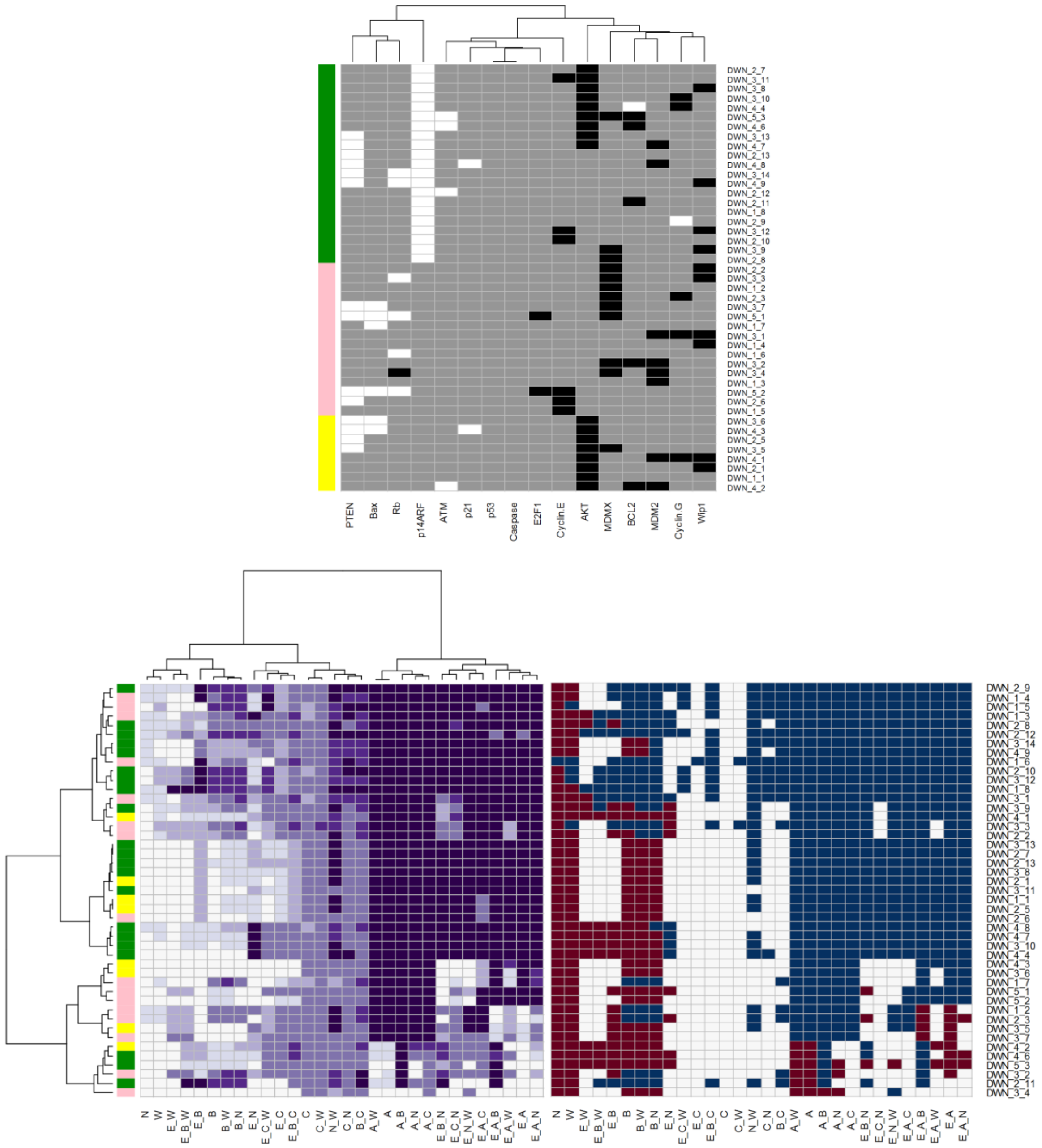

Supplementary Figure 6. Static network subtypes are not sufficient for predicting drug response. The 45 differentially wired networks (DWNs) were clustered into 3 groups by a measure of topological similarity, based on correlation between networks that are characterized by specific combinations (signatures) of the networks component (upper panel). However, networks of similar topology subtype do not necessarily cluster in terms of drug perturbation response profiles and major cellular phenotype (for drug efficacy: a gradient of purple colors; for major cellular phenotype: cell-proliferation-dominant (red), cell cycle arrest-dominant (white), cell death-dominant (blue)) (lower panel).

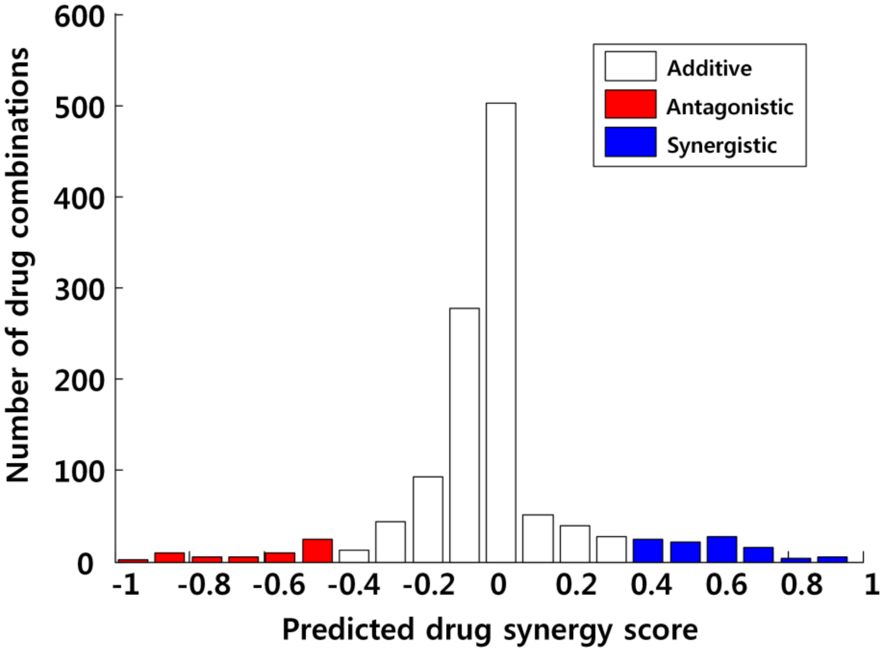

Supplementary Figure 7. Distribution of drug synergy score. We calculated the S scores for a total of 1,000 drug pairs and plotted the distribution, which follows a normal distribution. We subsequently converted the distribution to z scores to facilitate comparison and selection of synergistic/antagonistic thresholds. Based on the distribution, we defined drug pairs that have z scores of $z > 1.645$ or $z < -1.645$ as having significant synergistic or antagonistic effects.

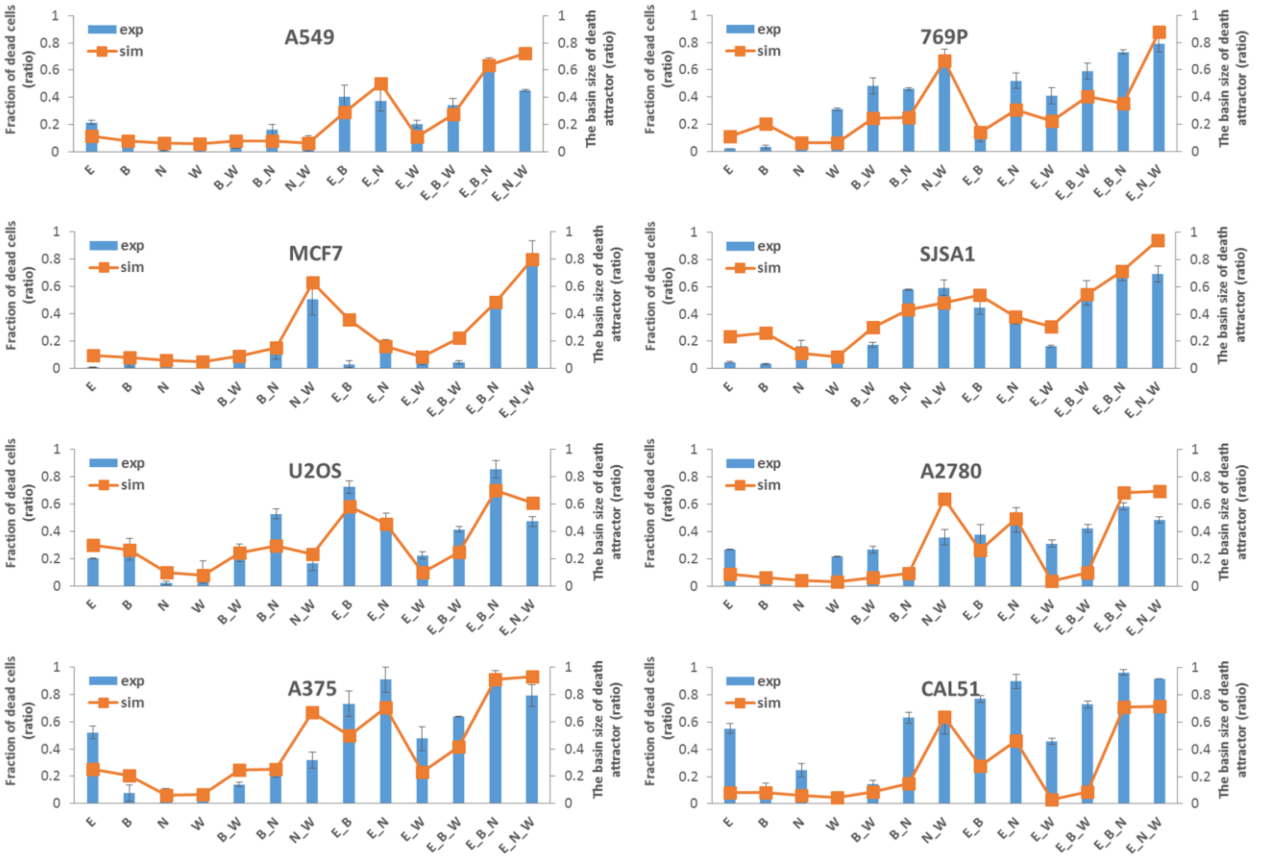

Supplementary Figure 8. Comparison of the ratio of cell death acquired from the perturbation analysis and time-lapse imaging experiments under various drug treatment conditions. The blue bars denote data of cell death ratio acquired from experiment and the orange lines denote the simulation results. The error bars indicate the standard deviation of experiments. In general, the simulation and experimental results showed similar response profiles across the different cell lines and treatment conditions.

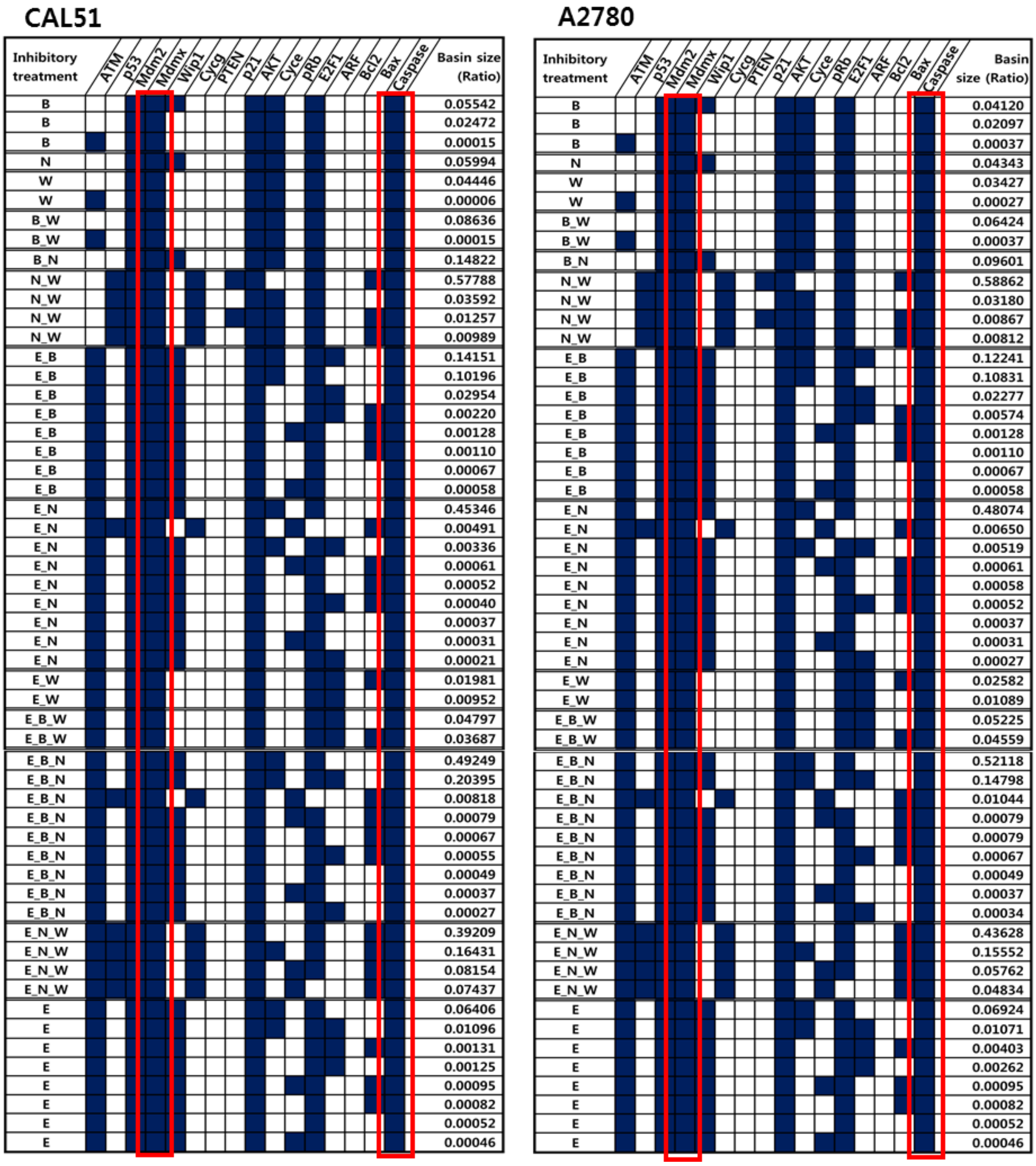

Supplementary Figure 9. Cell death attractor states and their basin size (ratio) under each different perturbation in CAL51 (left) and A2780 (right) specific networks**.** Our simulation results showed very similar perturbation responses for A2780 and CAL51 (Supplementary Fig. 8). Both networks share two alterations including constantly inactivated PTEN and constantly activated AKT (PTEN(I) and AKT(A)). MDMX, which is not constantly activated in CAL51 but in A2780, is the only difference in these two cell line specific networks. To examine the effect of constantly activated MDMX (MDMX(A)), we investigated the attractor states of A2780 and CAL51 and found that both networks have same cell death attractor states, although each basin of attraction is slightly different. This result indicates that in the presence of specific node alterations where PTEN is constantly inactivated and AKT is constantly activated, a constantly activated MDMX does not significantly affect the cell death ratio that is determined by the overall network dynamics

in our model. MDMX is all “ON” at the attractor states of CAL51 model, even though it is not constantly activated in CAL51 specific network. Each attractor state is represented by a set of 16 boxes in each row. Each box represents the state of each network node at the attractor state and a navy box or white box indicates “ON” or “OFF”, respectively. Two long red boxes mean MDMX and Caspase.

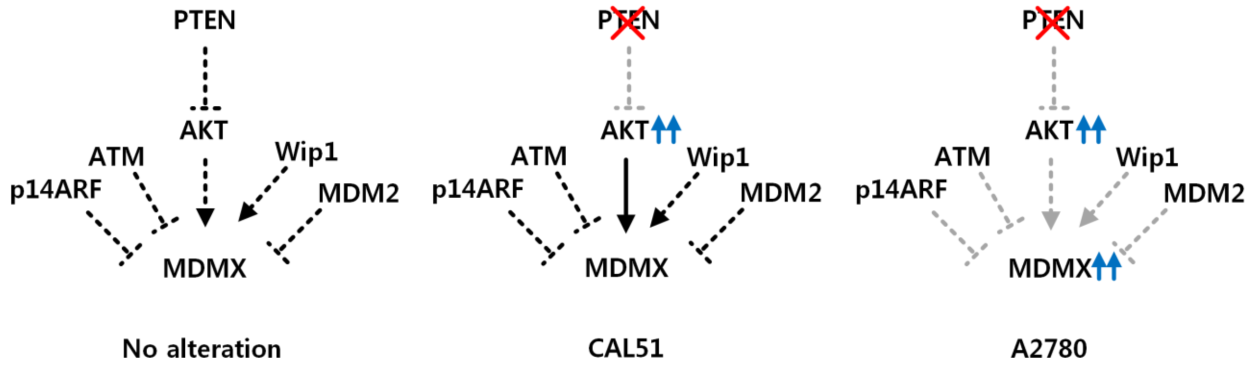

Supplementary Figure 10. PTEN-AKT-MDMX interactions in CAL51 and A2780 models. MDMX has two activating upstream regulators, AKT, Wip1 and three inhibitory upstream regulators, ATM, p14ARF and MDM2 in our model. In the absence of alteration, the state of MDMX is determined by combination of five inputs (each interaction is denoted by black dotted line). But, in the presence of node alterations where PTEN is constantly inactivated and AKT is constantly activated (in CAL51 model), a constantly activated AKT becomes a dominant input that keeps MDMX "ON" even though MDMX has multiple upstream regulators. Therefore, a constantly activated MDMX in A2780 does not significantly affect the cell death ratio determined by the overall network dynamics in our model. The black solid line represents a constantly activated link and gray dotted lines denote insignificant links as they are from constantly inactivated nodes or their target is constantly activated or inactivated nodes.
