# Supplementary material for: Network dynamics-based cancer panel stratification for systemic prediction of anticancer drug response
Source: Nat Commun. 2017 Dec 5;8:1940. doi: 10.1038/s41467-017-02160-5 (PMC5717260; doi:10.1038/s41467-017-02160-5)
Supplement: Supplementary file 3 — Description of Additional Supplementary Files [file 41467_2017_2160_MOESM3_ESM.pdf]

Description of Additional Supplementary Files

File Name: Supplementary Data 1
Description: Genomic features of 83 human cancer cell lines from the Cancer Cell Line Encyclopedia and construction of the differentially wired networks (DWNs)

File Name: Supplementary Data 2
Description: Drug perturbation response profiles of the 45 DWNs

File Name: Supplementary Data 3
Description: Categorization of drug response by major response phenotype, critical target(s) and critical determinant(s)

File Name: Supplementary Data 4
Description: Quantitative score of cellular response to perturbation (R score, D score, and S score)

File Name: Supplementary Data 5
Description: Simulation results and experimental data measuring cell death

File Name: Supplementary Data 6
Description: All the state transition logics for Boolean network modelling
